# Genomic basis of ecological niche divergence among cryptic sister species of non-biting midges

**DOI:** 10.1186/1471-2164-14-384

**Published:** 2013-06-10

**Authors:** Hanno Schmidt, Bastian Greshake, Barbara Feldmeyer, Thomas Hankeln, Markus Pfenninger

**Affiliations:** 1 Molecular Ecology Group, Biodiversity and Climate Research Centre (BiK-F) by Senckenberg Gesellschaft für Naturforschung and Goethe University, Biocampus Siesmayerstraße, Frankfurt am Main, 60054, Germany; 2 Institute of Molecular Genetics, Biosafety Research and Consulting, Johannes Gutenberg-University, Becherweg 30a, Mainz, 55128, Germany; 3 Current address: Department of Evolutionary Biology, Johannes Gutenberg-University, Johannes-von-Müller-Weg 6, Mainz, 55128, Germany

**Keywords:** Adaptive sequence evolution, Positive selection, McDonald-Kreitman test, *Chironomus riparius*, *Chironomus piger*

## Abstract

**Background:**

There is a lack of understanding the evolutionary forces driving niche segregation of closely related organisms. In addition, pinpointing the genes driving ecological divergence is a key goal in molecular ecology. Here, larval transcriptome sequences obtained by next-generation-sequencing are used to address these issues in a morphologically cryptic sister species pair of non-biting midges (*Chironomus riparius* and *C. piger*).

**Results:**

More than eight thousand orthologous open reading frames were screened for interspecific divergence and intraspecific polymorphisms. Despite a small mean sequence divergence of 1.53% between the sister species, 25.1% of 18,115 observed amino acid substitutions were inferred by α statistics to be driven by positive selection. Applying McDonald-Kreitman tests to 715 alignments of gene orthologues identified eleven (1.5%) genes driven by positive selection.

**Conclusions:**

Three candidate genes were identified as potentially responsible for the observed niche segregation concerning nitrite concentration, habitat temperature and water conductivity. Additionally, signs of positive selection in the hydrogen sulfide detoxification pathway were detected, providing a new plausible hypothesis for the species’ ecological differentiation. Finally, a divergently selected, nuclear encoded mitochondrial ribosomal protein may contribute to reproductive isolation due to cytonuclear coevolution.

## Background

A decade-long debate reasons to what extent Darwinian selection or neutral processes are driving the molecular evolution of genes and thus the ecological divergence of species [1]–[4]. Selectionists argue that a large fraction of those non-synonymous DNA base substitutions in coding genes going to fixation should be driven by positive Darwinian selection [5]. Under a strict neutralist’s view, most fixed amino acid substitutions have no effect on fitness [6,7], because purifying selection constantly removes alleles with strongly deleterious effects on fitness while positive effects were thought to be extremely rare. Later, the nearly neutral theory acknowledged that also substitutions with slightly deleterious effects may drift to fixation under realistic demographic scenarios [8,9]. While the neutral theory was the prevailing model for several decades, the comparison of whole genome sequences has recently produced evidence for an important role of natural selection [3]. In particular, for *Drosophila* species, natural selection has been shown to shape both the coding and non-coding parts of the genome [10,11]. However, before being able to draw general conclusions on the importance and mode of selection in shaping ecological divergence, more studies of systematically diverse taxa with differing life histories, demographies and mating systems are clearly needed [3]. The recent progress in sequencing technology [12,13] and resultant ability to sequence whole transcriptomes or genomes even for non-model species now opens up this opportunity [14]. Moreover, such approaches allow at the same time pinpointing the genomic basis of ecologically relevant traits and their evolutionary history [15]–[19]. This may be accomplished in two different ways: Based on known ecological differences of the taxa under scrutiny it is possible to assess the processes driving the evolution of genes likely associated with the relevant traits. This is an extension of the classical candidate gene or top down approach. In addition, scanning coding genes for positive selection allows for a bottom up approach, which Li *et al.*[20] called “reverse ecology”. The principle of the latter is to identify loci whose divergence was driven by positive selection and to infer hypotheses about ecological differences from their biological function. In both cases, however, it remains challenging to functionally link the identified patterns with observed phenotypic differences.

To contribute to this scientific debate, we have conducted a comparative analysis of larval transcriptomes among the dipteran midge sister species pair *Chironomus riparius* Meigen 1804 (synonym *C. thummi*, respectively *C. thummi thummi*) and *Chironomus piger* Strenzke 1959 (synonym *C. thummi piger*) [21]. As co-occurring, morphologically cryptic sister species, they are particularly interesting to perform comparative genomic analyses of ecological niche differences for several reasons. First, due to the shared evolutionary history, their general ecological niche is usually similar, which makes them prime candidates for interspecific competition and, to allow coexistence in sympatry, niche segregation [22]. Second, fixed genetic differences among them must have evolved during or after divergence, thereby also reflecting the selective forces leading to the observed ecological differences and/or reproductive isolation. Third, their coding gene sequences will almost certainly be sufficiently similar to distinguish orthologous from paralogous loci [23]. Fourth, the short evolutionary distance assures that the incidence of multiple mutational hits at individual sites is negligible, making it possible to infer which mutational changes have occurred since speciation [24]. Fifth, as morphologically cryptic species, identified differences likely not involve anatomic differences, thereby reducing the complexity of associating gene evolution with phenotypic differences.

The two *Chironomus* species show differential swarming behaviour in the field, acting as a prezygotic isolation mechanism [25]. While some studies indicate that *C. riparius* and *C. piger* readily form viable and fully fertile interspecific hybrids in the laboratory [26], others estimate fertile hybrids in the wild to be effectively absent, due to fertility reductions caused by hybrid dysgenesis syndromes [27]. Indeed, no ongoing hybridisation in the field could be found in an early chromosomal study [28], which was corroborated by a field survey applying microsatellites and mitochondrial markers [29]. Larvae of both species are widely distributed in small streams, ditches, ponds and puddles throughout the Holarctic [28]. The species are often dominating the local *Chironomus* larval community [30]. They frequently occur together at the same sites, however, usually one species prevails at a particular site [28,29]. As chironomids spend most of their lifetime as larvae and usually even do not feed as imagines during their few days in this stage [31], the larval stage is in general regarded as the relevant life cycle stage for ecological studies [32]. This is especially true for the two species under scrutiny, *C. piger* and *C. riparius*, as they show clear ecological differentiation as larvae [29] but nearly none as imagines [25]. Field data indicate that *C. piger* larvae are found preferentially in puddles and shallow ditches with higher maximum water temperatures, higher salinity, in particular higher nitrite and calcium concentrations as compared to sites inhabited by *C. riparius*[29]. The latter species often inhabits sediments with high organic content, indicating higher tolerance to anaerobic conditions [33], which was confirmed in different experimental studies [34,35]. In a recent experimental study *C. piger* coped better with higher nitrite concentrations [36]. Contrary to expectations from field studies, *C. riparius*’ fitness tended to be higher at both higher constant temperatures and larger daily temperature ranges. However, the interaction of both stressors favoured *C. piger* in warm, high nitrite habitats, thus concurring to the field observations [36]. Based on this previous knowledge on ecological niche differences, proteins with functions in cell respiration as well as those concerning response to temperature and solved ions, especially nitrite detoxification, are promising candidates for interspecific differences driven by positive selection.

In this study, the following three questions were thus addressed: i) Is positive Darwinian selection a major evolutionary driving force for the divergence of the sister species? ii) Can genes with signs of positive selection be conclusively linked to known ecological differences between the two sister species? iii) Can we derive hypotheses on yet unrecognised ecological differences between the sister species from the observed pattern of divergence?

## Results

### Sequencing, assembly and annotation

Sequencing the larval transcriptomes of the two midge species with the Roche 454 technique resulted in 2,123,605 quality filtered reads in total (Table 1). Assembly using the CLC Genomics Workbench yielded 42,524 contigs for *C. piger* and 29,917 for *C. riparius*, respectively (Table 1). Mean contig length was 621 bp for *C. piger* and 732 bp for *C. riparius*. N50 length was 805 bp for *C. piger* and 914 bp for *C. riparius*. Ninety-three percent (*C. piger*) and 94% (*C. riparius*) of all reads could be mapped against the obtained contigs.

**Table 1:** Summary statistics of transcriptome sequencing

	***C. piger***	***C. riparius***
Number of 454 reads	1,235,393	888,212
Number of contigs	42,524	29,917
Contig N50 length (bp)	805	914
Number of BLASTx hits < 1e^-10^	11,326	9,187
Number of unigenes	6,323	5,705

Using the BLASTx algorithm, 11,326 contigs of *C. piger* and 9,187 contigs of *C. riparius* matched entries in the Swiss-Prot database with e-values below the threshold of 1e^-10^. Merging hits with identical descriptions irrespective of the hits’ taxon assignment resulted in 6,323 unigenes in *C. piger* and 5,705 unigenes in *C. riparius* with an overlap of 4,738 genes. Of all contigs with BLASTx hits below 1e^-10^ a total of 9,527 could be annotated with GO terms (Additional file 1).

### Alignment quality and sequence divergence

**Figure 1:**
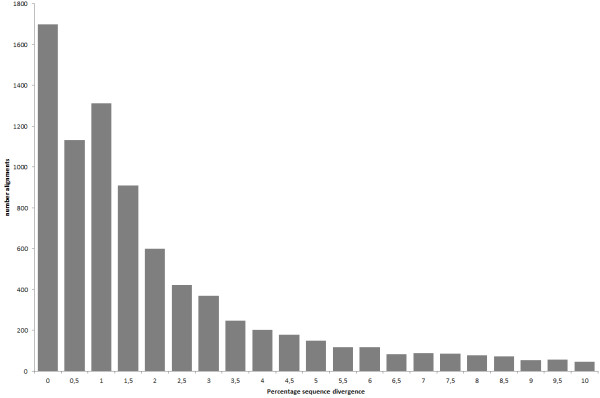
**Distribution of uncorrected sequence divergence between transcribed genes of *C. piger *and *C. riparius*.** Shown are the rates of substitutions between the two chironomid species per alignment. Alignments with more than 10% sequence divergence were not taken into account.

The programme ORFPredictor assigned open reading frames (ORFs), matching the set requirements, for 41,489 contigs in *C. piger* (98%) and for 29,391 contigs in *C. riparius* (98%), respectively. OrthoMCL built 12,685 groups of putative orthologues from translated sequences of the two sets of ORFs from *C. piger* and *C. riparius*. As 2,302 groups only contained sequences from one or the other species, a total of 10,383 groups were kept. For each group the best-fitting sequences of the two species were then aligned and re-translated. Trimming of those alignments ended in 8,031 final alignments with an average length of 402 bp. Of those, 1,711 alignments (21.3%) had no differences on the nucleotide level. Besides that, there was an apparent peak around 1% sequence divergence between the species (Figure 1). The mean sequence divergence was 1.53% on the nucleotide level and 1.68% on the amino acid level, equalling 18,115 amino acid substitutions in total.

### Detection of intraspecific genetic variation

Single nucleotide polymorphism (SNP) identification in the species-specific mappings considered sequencing quality scores of the used and surrounding nucleotides, mapping depth and quantity of the rarer allele for quality control. SNP detection across the 42,524 contigs in *C. piger* identified 72,096 high quality diallelic SNP positions. After discarding all SNPs outside the defined high quality ORFs, 25,375 SNP sites remained. Of those, 13,434 (52.9%) were non-silent, and 11,941 (47.1%) were silent. The 29,917 contigs in *C. riparius* yielded 33,826 SNP positions in total, 12,032 of which were within a high quality ORF. At 6,720 SNP positions (55.9%) the two alleles coded for different amino acids, while 5,312 SNP sites (44.2%) were silent. Classification of SNPs according to the frequency of their rarer allele showed the ratio of non-synonymous to synonymous SNPs to decrease at higher allele frequency (Table 2).

**Table 2:** Number and frequency distribution of SNPs in the two sister species

	**Pn**	**Ps**	Pn*/Ps*
Low frequency SNPs	2,421	1,341	0.46
Moderate frequency SNPs	4,069	3,326	0.31
Common SNPs	13,666	12,586	0.28

SNPs were classified by the frequency of their rarer allele as follows: low frequency < 5%, moderate frequency 5-15%, and common 15-50%. Pn = non-synonymous SNPs, Ps = synonymous SNPs, Pn*/Ps* = ratio of non-synonymous to synonymous SNPs per non-synonymous and synonymous sites, respectively.

### Measure of sequence evolution for the whole transcriptome

The estimated average proportion of amino acid substitutions fixed by positive selection, α, amounted to 0.251 for the 8,031 alignments included. This estimate is significantly greater than zero with regard to the calculated confidence interval (95% C. I.: 0.192, 0.308). The analysis was repeated under exclusion of (1) low-frequency and (2) low+moderate-frequency SNPs to correct for slightly deleterious mutations. This procedure slightly elevated α to (1) 0.265 (C. I.: 0.206, 0.323) and (2) 0.324 (C. I.: 0.264, 0.378), respectively.

### McDonald-Kreitman tests

The McDonald-Kreitman test (MKT), a robust test for positive selection using substitution and polymorphism data, could be applied to 715 alignments showing non-zero values in all four categories of nucleotide changes. Of those alignments, 11 (1.5%) had a ratio of non-synonymous substitutions (D_n_) to synonymous substitutions (D_s_) significantly greater than the ratio of non-synonymous polymorphisms (P_n_) to synonymous polymorphisms (P_s_) with a FDR-corrected p-value ≤ 0.05, hence bore signs of positive selection between *C. piger* and *C. riparius*. Due to lacking BLASTx annotations in three of them, eight alignments with reliable signs of positive selection and available annotations remained (Table 3).

**Table 3:** Alignments with significant MKT values and their correlating ω values

**Cluster**	**BLASTx annotation**	**(Dn/Ds)/ (Pn/Ps)**	**ω interspecific**	**ω**	**ω**	**GO terms *Biological Process***	**Hypothesised relation to observed niche differences**
				***C. piger***	***C. riparius***		
rp4125	Plasma membrane calcium-transporting ATPase 3	9.24	0.12	**9.75**	0.08	metabolic process, calcium ion transmembrane transport, blood coagulation, ATP biosynthetic process, platelet activation	Tolerance of *C. piger* to increased CaCO_3_ levels due to effective removal of Ca-ions from cells.
rp4927	Dolichyl-diphosphooligosaccharide--protein glycosyltransferase subunit STT3A	9.86	0.24	x	x	protein N-linked glycosylation via asparagine	None
rp6124	Signal recognition particle receptor subunit alpha	9.31	0.20	x	**#**	intracellular protein transport, GTP catabolic process, SRP-dependent cotranslational protein targeting to membrane, axonogenesis, regulation of protein secretion	None
rp6180	1-acyl-sn-glycerol-3-phosphate acyltransferase gamma	9.21	0.49	1.00E-04	1.00E-04	metabolic process, phospholipid biosynthetic process	None
rp6592	Sulfide:quinone oxidoreductase, mitochondrial	9.68	0.28	1.00E-04	**20.37**	oxidation-reduction process	Presence of *C. riparius* in anoxic, organic sediments with increased H_2_S content due to effective detoxification.
rp7097	NADH-cytochrome b5 reductase 2	11.62	0.11	x	x	oxidation-reduction process, lipid biosynthetic process, sterol biosynthetic process	Tolerance of *C. piger* to increased nitrite levels due to effective transformation of methhaemoglobin to haemoglobin.
rp8023	39S ribosomal protein L44, mitochondrial	18.44	**1.10**	**3.77**	**1.12**	RNA processing	Reciprocal reproductive isolation due to cytonuclear incompatibilities.
rp8328	Calreticulin	10.62	0.45	x	x	protein folding, brain morphogenesis, central nervous system development, peripheral nervous system development, startle response, olfactory behavior, locomotion involved in locomotory behavior	Tolerance of *C. piger* to increased habitat temperatures.

Shown are values for all alignments with a significant MKT value (FDR-corrected p-value ≤ 0.05) and a significant BLASTx hit (1e^-10^) against the SwissProt database. ω > 1, indicating positive selection, printed in bold. x = incalculable due to missing data for the four-species alignments, # = infinite value.

(Dn/Ds)/(Pn/Ps) > 1 is an indicator of positive selection. Interspecific ω values are calculated with KaKs_Calculator using the same alignments as MKT; species-specific ω values are calculated with codeml using four-species alignments.

### Branch-specific ω

OrthoMCL built 19,800 groups of putative orthologous sequences by means of the four sets of protein sequences from *C. piger*, *C. riparius* and the two mosquitoes *A. aegypti* and *C. quinquefasciatus*. As only groups with at least one sequence per species were analysed further, a total of 4,232 groups were kept. Trimming of the re-translated alignments of the best-fitting sequences per species resulted in 2,558 final alignments with an average length of 496 bp. Using the programme PAML, signs of positive selection were found in 316 alignments (12%) in *C. piger* and 336 alignments (13%) in *C. riparius*.

Among the eight annotated genes with signs of positive selection in the MKT, two had branch-specific ω > 1 in one species and ω < 1 in the other, indicating directional selection. Here, plasma membrane calcium-transporting ATPase 3 showed signs of positive selection only in *C. piger* and mitochondrial sulfide:quinone oxidoreductase only in *C. riparius*. One protein (39S ribosomal protein L44, mitochondrial) showed ω larger than one in both species, indicating disruptive selection between the species. The rest were either incalculable in one or both species due to missing sequence data in the four-species alignments (4×) or had branch-specific ω smaller than one (1×).

## Discussion

Comparative sequence-based studies are highly dependent on data quality. The stringent approach taken here with orthology assignment of high quality contigs and rigorous trimming of the resultant alignments is therefore rather conservative, despite a considerable loss of data. One source of errors that necessitated substantial trimming of the data was homopolymer stretches that pose well-known problems when sequencing with the 454 Roche technology [12,37,38]. By only using SNPs with the rarer allele observed at least twice, a misidentification of SNPs due to sequencing errors should not have posed considerable problems as the overall sequencing error rate (including both, indels and base substitutions) is only 1.07% for 454 GS FLX [39]. With a mean coverage of 176 observed at the SNP positions used, this equals p = 0.02. Considering that only a small portion of those 454 sequencing mistakes are base replacement errors [39], the effective error rate is even lower. Furthermore, included errors will most certainly contribute proportionally more to non-synonymous than to synonymous SNPs due to the high ratio of non-synonymous to synonymous positions. The resulting overestimation of the ratio of non-synonymous to synonymous SNPs would therefore rather lead to a lowered, conservative signal of the impact of adaptive evolution in the MKT and the calculation of α.

It is, however, important to be aware of other immanent limitations of Next-Generation-Sequencing-generated transcriptomic data for population genetic analyses. Transcriptomic sequences might miss alleles due to allele specific gene expression [40]. This phenomenon is wide-spread [41,42] and may result in an underestimation of intraspecific variation. However, as allele specific gene expression is due to differential DNA methylation, this may not present a substantial problem here, as Dipterans have lost the main methylation enzymes in their evolutionary history [43]. Other limitations for the use of our data are due to the experimental design. The pooling of individuals, although a common and recommended strategy for non-model organism studies aiming to identify as many alleles as possible [44], did not allow scoring individual genotypes, which in turn precluded calculations of allele frequencies. For this study, 60 individuals per species were sequenced at a mean mapping depth of 9.9 nucleotides per position for *C. piger* and 9.5 for *C. riparius* across all loci. As we have used a minimum coverage of 10 reads per position in each species for SNP detection, we have discovered only segregating alleles with a frequency over 0.25 with a probability of > 90%.

### Interspecific patterns for the whole transcriptome

The mean interspecific nucleotide sequence divergence of 1.53% on the cDNA level, the mean silent site rate in coding regions (dS) of 0.06 and the fact that 1,711 alignments (21.3%) showed no differences at all, together illustrate the relatively close phylogenetic relationship between the sister species *C. piger* and *C. riparius*. Accordant calculations between two reproductively isolated ‘biotypes’ of the cryptic whitefly species *Bemisia tabaci* revealed a mean divergence of 0.83% [45], while 1.5% divergence were found between the two closely related *Drosophila* species *D. simulans* and *D. sechellia*[46] and 2.5% divergence between two very young sympatric crater lake cichlid fishes [47]. A recent study across transcriptomes of the copepod *Tigriopus californicus* even revealed higher median divergence on the interpopulation level [2.71%, 17]. On the other hand, the conservative alignment strategy taken here, with exclusion of seemingly too heterogeneous parts, may have led to a slight underestimation of the overall sequence divergence, as suggested by the 2.14% of coding sequence divergence in the vwvz/7B globin gene cluster of *C. piger* and *C. riparius*[48].

Since estimations of the species divergence time of *C. piger* and *C. riparius* based on large amounts of sequence data are lacking to date, the overall synonymous substitution rate of the ORFs was used for a rough molecular clock calculation. Drosophilids are the best-analysed dipteran system concerning molecular clock analyses with rates of synonymous substitution per synonymous site per million years of 0.016 [49], 0.015 [50], and 0.011 [51]. Transferring these brachyceran rates to the nematocerans *C. piger* and *C. riparius* resulted in an estimation of the species splitting event about 1.3 to 1.8 million years ago, which translates to a multiple number of generations since divergence in this multivoltine species. This time frame clearly illustrates the existing potential for evolutionary processes to shape the species’ genomes.

### Number of genes identified

The number of genes found in the larval transcriptomes of *C. piger* (6,322) and *C. riparius* (5,705) is roughly a third to half the number of genes found in the genomes of the nematocerans *Anopheles gambiae* [n = 13,683, [52], *Aedes aegypti* [n = 15,419, [53], *Culex quinquefasciatus* [n = 18,883, [54] and the dipteran *Drosophila melanogaster* [n = 13,379, [55]. The discrepancy is explained by the fact that certainly not all genes are expressed in the L4 stage of the midges and only a single stressor (heat) was applied. For example, *Drosophila* larval stages were shown to express only 4,900 to 7,000 different mRNAs at once [56]. Moreover, using non-normalised cDNA libraries likely resulted in missing genes with low expression levels.

### Global patterns of gene evolution

The α statistics showed approximately a quarter (25.1%) of the 18,115 amino acid substitutions to be driven by positive selection. This finding is close to the average of 30.1% found by Eyre-Walker in a meta-study of 25 interspecies comparisons [5]. In another meta-study Fay reported that 47% of 38 analysed species showed signs of positive selection, often affecting ca. 40% of all non-synonymous substitutions [57]. Among other factors, the level of adaptive evolution was shown in the former study to positively correlate with effective population size [5]. Bayesian coalescence analysis of published mitochondrial COI sequences estimated the effective mitochondrial population size to be quite high with 1.8 × 10^7^ (c.f. 1.7 × 10^6^ – 8.0 × 10^7^) for *C. piger* and 3.1 x 10^6^ (c.f. 1.3 × 10^5^ – 1.4 × 10^7^) for *C. riparius*, respectively [29]. An average F_ST_ of 0.027 for *C. piger* and 0.046 for *C. riparius* indicates little among population differentiation in both species [29]. Drift within both species thus seems to be a minor driving force here, a circumstance rendering natural selection particularly effective [58].

### Inference of positive selection from polymorphism data

For the detection of positive selection on the level of individual genes, most reliable results can be gained from the MK test, which is more robust to demographic biases than, e.g. the popular dN/dS method [59,60]. However, the MK approach necessitated a more than 80% reduction of the data set, due to the high sequence similarity between the sister species. Of the remaining 715 genes used to calculate MK statistics, 11 (1.5%) showed significant signs of positive selection.

This proportion of 1.5% of positively selected candidate genes inferred with the MKT appears rather moderate. This is especially so, since the genes expressed in the final larval stage of chironomids should be particularly prone to increased diversifying selection compared to the rest of the genome, because this is the phase in the life of the midges during which the competition (e.g. for space and food) is potentially most severe among species [61]. A recent genomic comparison of congeneric shallow and deep sea urchins indeed confirms that in particular genes expressed in the ecologically divergent life cycle stage show increased signatures of positive selection [15]. The chironomid L4 transcriptomes may thus perhaps even overestimate the impact of adaptive evolution on the entire coding genome. On the other hand, the MKT is rather conservative and positive selection having occurred early in divergence may have lost its signature [62].

### Ecological implications of positively selected genes

Although a sign of positive selection alone is only the first step in defining the genetic basis of ecological differentiation, such analyses are able to deliver promising potential candidate genes [63]. Among the eleven genes with significant signs of positive selection in the MKT, eight could be functionally annotated. Five of these genes suggest a correlation either to observed or so far unrecognised ecological differences between *C. piger* and *C. riparius* or to the evolution of reproductive isolation.

One of them, calreticulin, is a chaperone involved in protein folding and rejection of misfolded proteins in the endoplasmic reticulum [64]. It might thus be linked to the different maximum temperatures in the respective habitats of *C. riparius* and *C. piger*[29], as increased temperature destabilises and degrades proteins [65].

The NADH-cytochrome b5 reductase 2 is also known as methemoglobin reductase, which describes its main function, the reduction of methaemoglobin to haemoglobin [66]. One of the main environmental agents causing the formation of detrimental methaemoglobin are nitrate/nitrite [67], thus matching the observed and experimentally confirmed differential adaptation of the chironomid sister species to environmental nitrite concentrations [29,36]. An increased efficiency due to positive selection in converting methaemoglobine would certainly explain the higher nitrite tolerance of *C. piger* and thus identify a genomic basis for the observed niche difference. Because chironomids possess more than 40 different globins [68,69] which can make up as much as 90% of the last instar’s total hemolymph protein [48,70], this might be of particular importance. Unfortunately, the methemoglobin reductase was not found in the dataset of all four taxa used in the branch-specific test, which precludes the inference of the direction of selection.

The branch-specific ω for plasma membrane calcium-transporting ATPase 3 (PMCA3) revealed positive selection on the *C. piger* branch (ω = 9.75), as opposed to a neutral evolution of this gene in *C. riparius* (ω = 0.08). PMCA3 is responsible for the removal of calcium out of the cell [71]. Interestingly, Pfenninger and Nowak [29] demonstrated conductivity in general and CaCO_3_ concentrations in particular to be significantly correlated to the relative abundance of *C. piger* and *C. riparius*, with the latter preferring lower concentrations, thus lending ecological plausibility to PMCA3 as a candidate gene for positive selection.

Besides proposing candidate genes for the known ecological differences between *C. riparius* and *C. piger*, the reverse ecology approach of studying whole transcriptomes also yields novel gene candidates with plausible relevance to the ecology and evolution of the sister taxa:

Sulfide:quinone oxidoreductase is involved in hydrogen sulfide detoxification [72]. Hydrogen sulfide is produced by microorganisms under anaerobic conditions, being highly toxic to most metazoans. The prevalence of *C. riparius* in anaerobic habitats like sewage sludge [34] might therefore at least partly be based on higher hydrogen sulfide tolerance. In accordance with this hypothesis is the high ω detected on the *C. riparius* branch (20.37) for sulfide:quinone oxidoreductase in contrast to the *C. piger* branch (1e^-4^), suggesting adaptation to increased H_2_S levels in sediments of eutrophic water.

The nuclear encoded 39S ribosomal protein L44 interacts closely with mitochondrially encoded rRNA molecules [73], thus providing the basis for cytonuclear coevolution, in which structural changes in one partner (usually the faster evolving mitochondrial genome) provide the stage for selection-driven compensatory changes in the interaction partner [74]. Intriguingly, the branch specific analysis shows positive selection along the branches of both species. If the resulting changes in protein structure led to incompatibilities with the mitochondrial rRNA of the respective other species in the formation of mitochondrial ribosomes, they may at least partially explain the observed reproductive isolation in the wild [75]. Cytonuclear incompatibilities indeed have been shown to confer reproductive isolation among other insects species [76].

## Conclusions

In accordance with recent meta-studies [5,57] on the genome-wide prevalence of positive selection in insects, the data presented here argue that such Darwinian processes are likely to have played an important role in the divergence of *Chironomus piger* and *C. riparius*. Several gene loci showing signatures of directional or disruptive positive selection can explain observed ecological characteristics of niche divergence. In addition, the reverse ecology approach pointed towards a new hypothesis with respect to the higher anaerobic tolerance of *C. riparius*. The signs of positive selection on a sulfide:quinone oxidoreductase gene in this species suggest the possibility that this adaptation depends at least partly on a higher H_2_S-tolerance.

Obviously, the above hypotheses, based solely on bioinformatic analysis of transcriptome sequences, need further investigations, in particular ecological experiments and functional protein assays, to functionally link the proposed candidate genes to the observed traits. In addition, there are likely several, if not many other fixed functional differences among the sister species, which affect protein structure and gene expression and thereby determine their respective niches. The presented study is therefore but a first step to fully understand the genomic basis of the ecological differences between *C. piger* and *C. riparius*. However, since many of the known ecological differences are plausibly mirrored by patterns of sequence evolution, the taken approach appears to be a valuable starting point to dig deeper into the genomic details of niche segregation.

## Methods

The *Chironomus riparius* and *C. piger* specimen used in this study were taken from laboratory cultures originally collected in 2009 from the field in south-western Germany as described in Nemec *et al.*[36]. The species were kept in relatively large (> 400 individuals) populations for about 3–4 generations prior to the present study. In a previous study, populations of this size kept for more than 10 generations under identical conditions showed no significant reduction of genetic diversity due to drift [77]. Freshly hatched larvae were reared at 20°C and 27°C in climate chambers with 16 hours lighting per day and fed *ad libitum* on commercial flake food for aquarium fish. The two different conditions were chosen because the resulting data was planned to be used for a study on differential gene expression and play a minor role here. On attaining larval stage four (L4), 30 larvae per treatment and species were shock-frozen at −80°C.

### RNA isolation, library preparation and sequencing

RNA isolation with Qiagen RNeasy Mini Kit (Qiagen) from the 30 whole larvae per treatment and species was followed by DNase digestion and purification of mRNA with Invitrogen Dynabeads (Invitrogen). Subsequently, four cDNA libraries, barcoded with multiplex identifier adaptors, were created using the cDNA Rapid Library Preparation Method Manual (Roche). Libraries were not normalized since this data was intended for use in a further gene expression study. After titration, each library was sequenced on half a titanium plate on a Roche 454 GS FLX system (Roche) according to the manufacturers’ instructions. All sequences were deposited in the NCBI Sequence Read Archive (accession numbers SRR834934, SRR835079, SRR834592, SRR834593). Sequences were processed to remove adaptors, low quality sequence parts (at least an average quality score of 20 for the last ten bp), and sequences below 50 bp prior to assembly.

### Assembly and annotation

Filtered reads were pooled per species and assembled *de novo* using the CLC Genomics Workbench 4.9 (CLC Bio, Aarhus, Denmark) with default settings (word size = 20, bubble size = 50). After assembly all reads were mapped back against the contig sequences for quality control and identification of variable sites (see below).

All obtained contigs were compared to the Swiss-Prot database using the BLASTx algorithm [78] with a cut-off E-value of ≤ 1e^-10^. Functional annotation was performed using the terminology provided by the Gene Ontology (GO) Consortium [79] in the category *Biological Process* (BP). This category “refers to a biological objective to which the gene or gene product contributes” [79] and therefore allows most informative linkage to ecological differences. The Swiss-Prot accession number of the best local alignment was used to annotate the respective gene with the associated GO terms. The Uniprot GO annotation file (v2.0) was obtained from the download section of geneontology.org.

#### Obtaining high quality alignments of orthologous sequences of C. piger and C. riparius

Protein sequences were obtained by predicting open reading frames (ORFs) with the Python program ORFPredictor (unpublished software by L. Wissler, available upon request by the authors). ORFPredictor uses the best local alignment from the BLASTx searches described above and searches the respective ORF from that starting point. For all contigs without BLAST hit, ORFPredictor outputs the longest contiguous closed ORF above the set minimum of 30 amino acids. Everything but the ORF sequences was discarded for downstream analyses. To obtain alignments of orthologous genes, the protein sequences were clustered using orthoMCL 2.0 [80] with standard settings. To remove paralogs, the sequences of all clusters with at least one sequence of each species were aligned using T-Coffee 8.99 [81]. For each cluster only the sequence of each species with the highest similarity to the sequence of the other species was used for further analyses. Pair-wise alignments were created using MUSCLE 3.8.31 [82] with standard settings. Those final protein alignments were (re)converted to the respective nucleotide alignments using PAL2NAL 13.0 [83], drawing on the respective nucleotide sequences after ORF-prediction. The nucleotide alignments were trimmed to the same length in both species. Visual inspection of the alignments showed the ends to frequently contain frame shifts, mainly due to differences in homopolymer length in 454 *Chironomus* sequences. Consequently, the nucleotide alignments were further trimmed by 15 bp at each end. As an additional quality control step, all alignments with a pair-wise p-distance > 0.1 between the two *Chironomus* species were excluded. This highly conservative filtering to remove residual poor alignments was chosen as it has been shown that even the intergenic regions of *C. piger* and *C. riparius* show an overall sequence identity of 90.8% [48].

### Counting polymorphic and divergent sites

Substitution counts were obtained from the output of KaKs_Calculator 1.2 [84], SNP detection was performed using the accordant tool in the CLC Genomics Workbench and the respective mappings of the aligned contigs. Only SNPs with a minimum mapping depth of ten, two alleles and at least two occurrences of the rarer allele were considered. Any SNP with a quality score below 20 and any with more than three surrounding bases with quality scores below 15 was ignored. SNPs then got classified as silent/ non-silent using self-written scripts. SNP-counts for both species were added up per gene.

### Measurement of sequence evolution for the whole transcriptome

Evidence for the transcriptome-wide prevailing mode of selection between two species can be gained by estimating α, the average proportion of interspecific amino-acid substitutions which are driven by adaptive evolution [85,86]. The calculation was performed using the method described by Smith and Eyre-Walker [86] and implemented in the DoFE software package 3.0 [87]. The analysis was based on the counts for non-synonymous and synonymous substitutions, non-silent and silent polymorphisms and non-synonymous and synonymous positions per gene of the 8,031 two-species alignments. The confidence interval for α was obtained by 1,000 bootstraps with randomly selected genes. As slightly deleterious mutations contribute more to polymorphisms than to divergence, the SNPs were then classified by the frequency of their rarer allele into low frequency SNPs (< 5%), moderate frequency SNPs (5-15%), and common SNPs (15-50%) following Fay *et al.*[88]. Afterwards, the calculation of α was repeated under exclusion of low frequency SNPs and low+moderate frequency SNPs, respectively.

### McDonald-Kreitman tests

The McDonald-Kreitman test [89] identifies patterns of sequence evolution by comparing synonymous/non-synonymous divergence versus silent/non-silent polymorphisms. It is very robust to different demographic factors [59,60] and does not suffer from inherent problems of ω analyses concerning nonlinear correlations of two randomly distributed variables [90]. The MKT itself depends on the assumption that D_n_/D_s_ > P_n_/P_s_ indicates an excess of adaptive amino acid substitutions and thus, positive selection. No input value must be zero. The distribution across the four classes is then tested for independence on a 2 × 2 contingency table using an appropriate statistic (χ^2^ test with false discovery rate (FDR) correction for this study).

### Inferring the direction of positive selection

To distinguish whether positive selection acted directionally on the branch leading to the one or the other species for the genes identified by MKT, a likelihood approach depending on phylogenetic alignments was used. Protein sequences from the phylogenetically closest genome sequenced organisms *Aedes aegypti* (PEPTIDES-AaegL1.2) and *Culex quinquefasciatus* (PEPTIDES-CpipJ1.2) were downloaded from VectorBase (vectorbase.org). The predicted ORFs for *C. piger* and *C. riparius* specified above were also used for the subsequent analyses. Clusters obtained by OrthoMCL as described above were split into two groups, according to phylogenetic relatedness: *C. riparius* and *C. piger* as non-biting midges and *A. aegypti* and *C. quinquefasciatus* as biting midges. Pair-wise MUSCLE alignments of these sub-clusters were then merged with the same program and re-translated as described above, additionally using the nucleotide sequences of *C. quinquefasciatus* (TRANSCRIPTS-CpipJ1.2) and *A. aegypti* (TRANSCRIPTS-AaegL1.2) from VectorBase. Trimming was performed as described in the upper part.

Tests on positive selection were performed by applying the codeml algorithm from the PAML program package 4.4 [91,92] to the four-species alignments. Some models in codeml allow discrete ω values for different taxa in one analysis, providing valuable information about the species’ contribution to divergent evolution. Calculation of global ω values using the one ratio model was followed by calculation of branch-specific ω using the free ratio model. As the free ratio model is very parameter-rich and therefore prone to biases, only alignments with significantly better likelihood scores (χ^2^, p = 0.05) for the more sophisticated free ratio model were considered for subsequent summary. Alignments with dS > 1 on the chironomid branches were discarded.

## Competing interests

The authors declare that they have no competing interests.

## Authors’ contributions

HS carried out the experiments and data analysis, participated in laboratory and bioinformatics work and drafted the manuscript. BG carried out bioinformatics work from orthology assignment to final alignments. BF participated in laboratory work and the study’s design and coordination. TH conceived the study, its design and coordination and helped to draft the manuscript. MP conceived the study, its design and coordination and drafted the manuscript. All authors read and approved the final manuscript.

## Supplementary Material

Additional file 1 **GO annotation of transcripts.** Representation of the GO terms from the category *Biological Process* across the Blastx annotated transcripts. Percentages are based on the number of genes successfully annotated per species. Shown are all GO terms associated to at least 1.5% of all transcripts. Click here for file
