# Supplementary material for: Genomic basis of ecological niche divergence among cryptic sister species of non-biting midges
Source: BMC Genomics. 2013 Jun 10;14:384. doi: 10.1186/1471-2164-14-384 (PMC3685581; doi:10.1186/1471-2164-14-384)
Supplement: Additional file 1 — GO annotation of transcripts. Representation of the GO terms from the category Biological Process across the Blastx annotated transcripts. Percentages are based on the number of genes successfully annotated per species. Shown are all GO terms associated to at least 1.5% of all transcripts. [file 1471-2164-14-384-S1.docx]

**Supplement 1: GO annotation of transcripts.**

Representation of the GO terms from the category *Biological Process* across the Blastx annotated transcripts. Percentages are based on the number of genes successfully annotated per species. Shown are all GO terms associated to at least 1.5% of all transcripts.

**
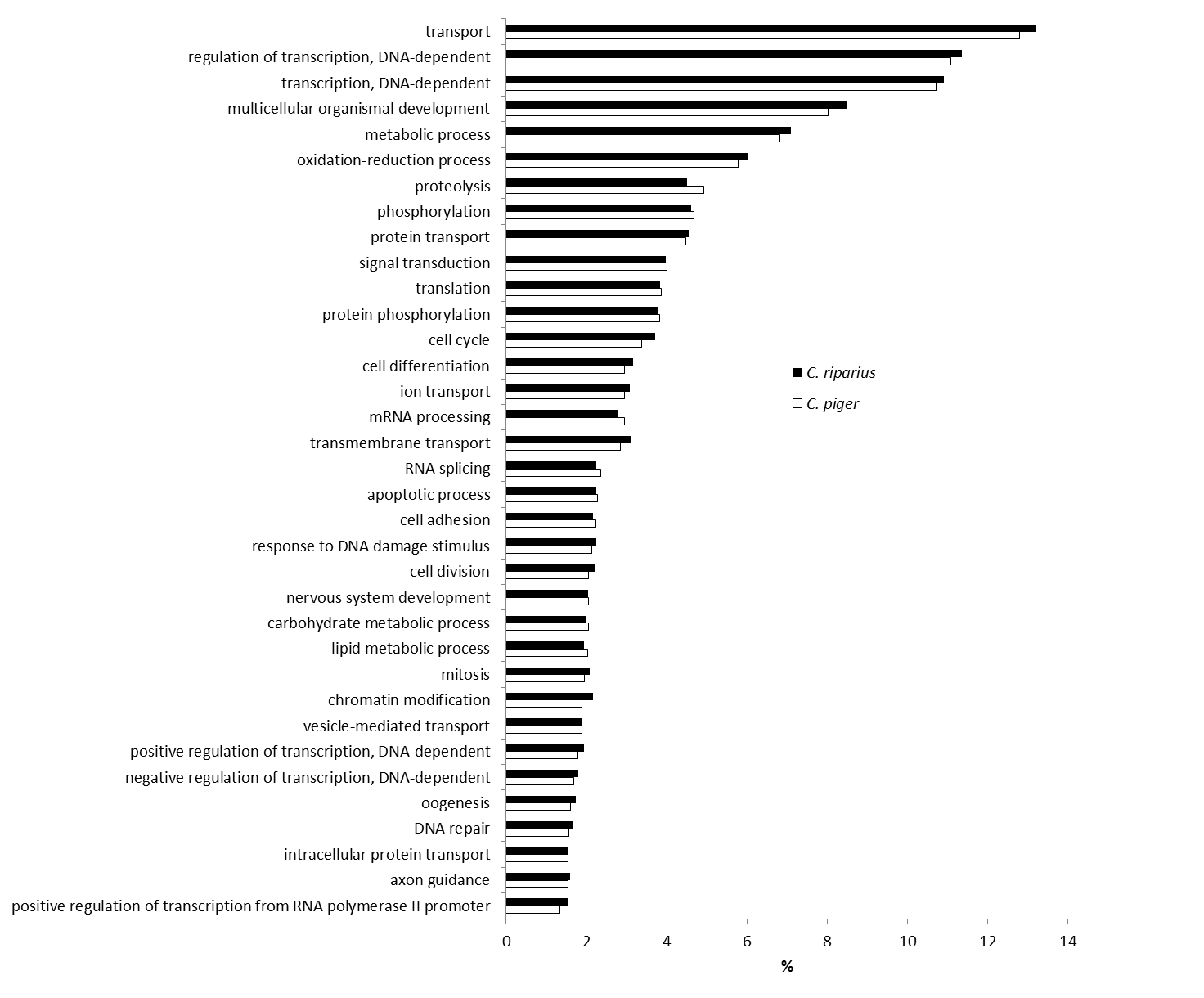
**
